# Surgical Treatment of Neuropathic Chronic Postherniorrhaphy Inguinal Pain: A Systematic Review and Meta-Analysis

**DOI:** 10.3390/jcm13102812

**Published:** 2024-05-10

**Authors:** Esmee Kwee, Mirte Langeveld, Liron S. Duraku, Caroline A. Hundepool, Michiel Zuidam

**Affiliations:** 1 Department of Plastic, Reconstructive Surgery and Handsurgery, Erasmus Medical Center, P.O. Box 2040, 3000 CA Rotterdam, The Netherlands; e.kwee@erasmusmc.nl (E.K.);; 2 Department of Plastic, Reconstructive Surgery and Handsurgery, Amsterdam University Medical Center, 1105AZ Amsterdam, The Netherlands

**Keywords:** inguinal hernia, postherniorrhaphy inguinal pain, neuropathic pain, surgical treatment, neurectomy, targeted muscle reinnervation

## Abstract

**Background/Objectives**: Neuropathic chronic postherniorrhaphy inguinal pain (CPIP) is a serious adverse outcome following inguinal hernia repair surgery. The optimal surgical treatment for neuropathic CPIP remains controversial in the current literature. This systematic review aims to evaluate the effectiveness of various surgical techniques utilized to manage neuropathic CPIP. **Methods**: The electronic databases Medline, Embase, Web of Science, Cochrane Central, and Google Scholar were searched. Inclusion criteria were defined to select studies reporting on the efficacy of surgical interventions in patients with neuropathic CPIP. The primary outcome was postoperative pain relief, as determined by postoperative numerical or nonnumerical pain scores. **Results**: Ten studies met the inclusion criteria. Three surgical techniques were identified: selective neurectomy, triple neurectomy, and targeted muscle reinnervation. Proportions of good postoperative results of the surgical techniques ranged between 46 and 88 percent. Overall, the surgical treatment of neuropathic CPIP achieved a good postoperative result in 68 percent (95% CI, 49 to 82%) of neuropathic CPIP patients (*n* = 244), with targeted muscle reinnervation yielding the highest proportion of good postoperative results. **Conclusions:** The surgical treatment of neuropathic CPIP is generally considered safe and has demonstrated effective pain relief across various surgical techniques. Targeted muscle reinnervation exhibits considerable potential for surpassing current success rates in inguinal hernia repair surgery.

## 1. Introduction

Inguinal hernia repair is one of the most frequently performed surgical interventions, with over twenty million yearly procedures worldwide [1]. Chronic postherniorrhaphy inguinal pain (CPIP) is a serious adverse outcome in hernia repair surgery. It has been defined by the International Association for the Study of Pain (IASP) as inguinal pain lasting for at least three months post-inguinal hernia repair [2]. CPIP has an estimated prevalence of 10 to 12 percent and influences normal daily activities in 0.5 to 6 percent of CPIP patients [3,4]. The symptomatology of CPIP is complex and depends upon the type(s) of pain that the patient is experiencing neuropathic or non-neuropathic pain [5].

Neuropathic CPIP is characterized by an activity-induced sharp pain, localized or radiating towards the groin and inner thigh. Symptoms of neuropathic CPIP include paresthesia, hypoesthesia, and hyperesthesia [5,6,7]. Neuropathic CPIP can be caused by intra- or postoperative injury to the inguinal nerves, primarily to the ilioinguinal, iliohypogastric, and/or genitofemoral nerve. Intraoperative nerve injury can result from surgical manipulation, thermal damage, or entrapment in tacks, sutures, or fixations [6,7]. Postoperatively, nerve injury may occur due to nerve compression through scar formation or involvement in a meshoma [6,7]. Although neuropathic pain is believed to account for approximately fifty percent of CPIP patients, precise prevalence rates of neuropathic CPIP remain uncertain in the current literature [7].

Conservative treatment for neuropathic CPIP involves pharmacological and interventional treatment modalities [3,4,5,6]. Surgical treatment may be considered if neuropathic CPIP is refractory to conservative measures. The current predominant surgical technique involves the selective or triple neurectomy of the ilioinguinal, iliohypogastric, and genitofemoral nerves, with or without concurrent mesh removal [8]. Previous studies have researched postoperative outcomes in patients experiencing chronic pain following hernia repair surgery [6,9,10]. However, the existing literature has not yet provided a comprehensive review examining surgical treatment options for neuropathic CPIP. Considering the significant worldwide incidence of neuropathic CPIP and its impact, identifying and implementing optimal treatment approaches is essential to reduce pain and improve functional ability. This systematic review aimed to identify the surgical techniques utilized in managing neuropathic chronic postherniorrhaphy inguinal pain, to evaluate the effectiveness of these surgical techniques, and to distinguish whether one surgical technique is the superior treatment option.

## 2. Materials and Methods

### 2.1. Literature Search

The methods and results of this systematic review are written following the Preferred Reporting Items of Systematic Reviews and Meta-Analyses (PRISMA) statement [11]. The electronic bibliographic databases Medline, Embase, Web of Science, Cochrane Central, and Google Scholar were searched from inception to 20 October 2022. The full electronic search strategy, including the search terms, is detailed in the Appendices (Appendix A).

### 2.2. Study Selection

Two authors (EK, ML) independently screened relevant studies based on titles and abstracts. Next, two authors screened and selected full-text articles (EK, ML) to meet the following inclusion criterion: clinical studies reporting the efficacy of surgical interventions in patients with neuropathic CPIP. Reviews, case reports, animal studies, conference abstracts, poster presentations, and non-English articles were excluded. Any discrepancies were resolved by consulting a third author (CH).

### 2.3. Data Extraction and Quality Scoring

During the data collection process, two authors (EK, ML) analyzed the included articles in detail and extracted data using a standardized data collection form. Again, any discrepancies were resolved by consulting a third author (CH). The following data were extracted: year of publication, publication type, sample size, hernia repair technique, proportion and percentage of patients with neuropathic CPIP, surgical treatment technique used in neuropathic CPIP management, reported outcomes, and time to follow-up. The primary outcome was the proportion of neuropathic CPIP patients who achieved good postoperative results. As pain assessment relies mainly on subjective measurements using a variety of scoring methods, this standardized primary outcome permits comparison of study outcomes. A good postoperative result was defined as a complete resolution of neuropathic pain, mild postoperative pain, or ‘significant pain relief’. Furthermore, a postoperative visual analog scale/numeric rating scale of 3 or less was considered a good result. For numeric rating scales of 0 to 3, a postoperative numeric rating scale of 1 or less was considered a good result. The secondary outcome was the proportion of patients that were pain-free postoperatively.

The two authors (EK, ML) classified the articles by strength of evidence using the Jovell and Navarro-Rubio classification (Appendix B) [12]. Quality assessment was performed using the study quality assessment tools of the National Institutes of Health (Appendix C) [13]. If any discrepancies occurred, a third author (CH) was consulted for resolution.

### 2.4. Statistical Analysis

For this study, we conducted a random-effects meta-analysis to statistically combine the proportions of patients with good postoperative results. We aimed to generate an overall pooled proportion for each surgical technique along with a 95 percent confidence interval. The meta-analysis was carried out in R using a generic inverse variance approach without Hartung–Knapp adjustments for estimates and confidence intervals. In this model, studies were weighted based on the inverse of the variance of the effect estimate. The comparison focused on the proportions of good postoperative results between selective neurectomy and other surgical techniques. Significance was established at a *p*-value of 0.05. The meta-analysis results are presented in a forest plot.

## 3. Results

**Figure 1:**
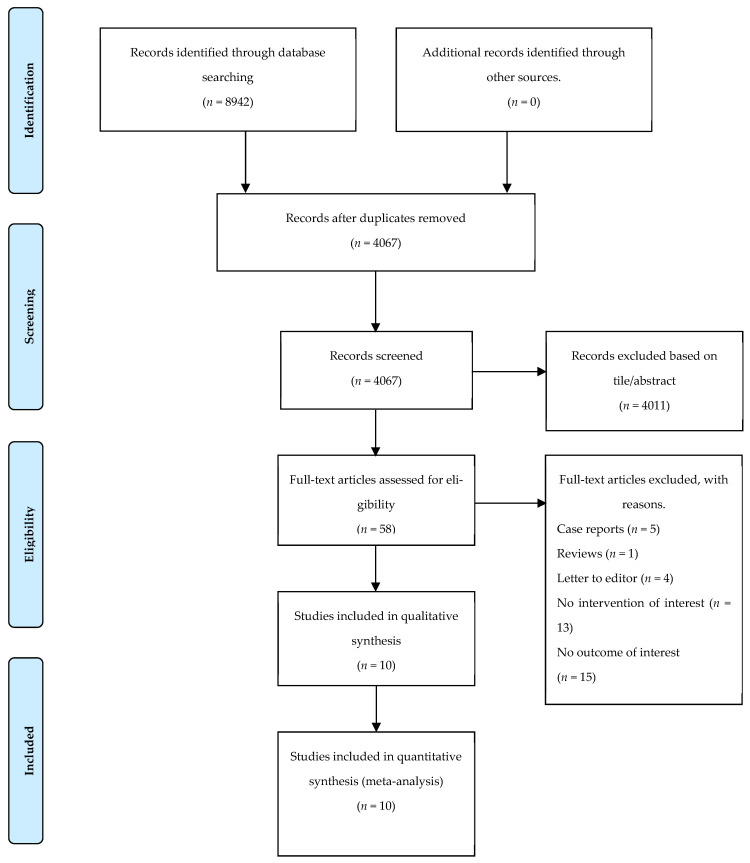
Flowchart regarding the selection of included articles according to the PRISMA standards.

Out of 8942 articles initially identified in the literature search, 4067 remained following the elimination of duplicates. Subsequent analysis led to the inclusion of ten articles meeting the inclusion criteria for this review (Figure 1) [14,15,16,17,18,19,20,21,22,23].

**Figure 2:**
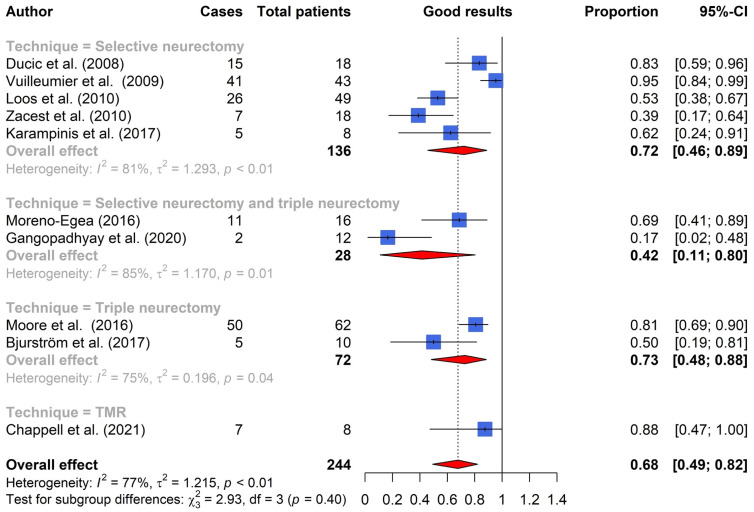
Pooled proportions of patients with good postoperative results per surgical technique [14,15,16,17,18,19,20,21,22,23].

The results of the included studies are presented in Table 1 and Figure 2. Among the selected studies, five specifically addressed neuropathic pain following inguinal hernia repair [15,16,20,21,22]. The remaining studies also provided information on neuropathic pain post other surgical procedures within the inguinal region, such as appendectomies and hysterectomies [14,17,18,19,23]. Whenever possible, outcome data were extracted and presented exclusively for patients undergoing treatment for neuropathic pain following inguinal hernia repair. Three studies did not distinguish outcomes between hernia repair and other surgeries within the inguinal region [17,19,23].

The literature outlines three surgical techniques for treating neuropathic CPIP: selective neurectomy, triple neurectomy, and targeted muscle reinnervation.

### 3.1. Selective Neurectomy

Selective neurectomy involves the surgical excision of the nerve(s) directly correlated with the neuropathic pain experienced—either the ilioinguinal, iliohypogastric, and/or genitofemoral nerve. This approach, detailed in five studies, exhibited an overall good postoperative result in 72 percent (95% CI, 46 to 89%) of neuropathic CPIP patients [14,15,16,17,18]. The ilioinguinal nerve was the most frequently excised nerve, accounting for approximately 63 percent of excised nerves, followed by the genitofemoral nerve at 24 percent and the iliohypogastric nerve at 13 percent.

### 3.2. Triple Neurectomy

Triple neurectomy entails the excision of all three inguinal nerves, demonstrating an overall good postoperative result in 73 percent (95% CI, 48 to 88%) of neuropathic CPIP patients, across two studies [21,22].

### 3.3. Selective and Triple Neurectomy

Selective and triple neurectomy were discussed in two studies without separate outcome presentations [18,19]. Overall, they yielded a good postoperative result in 42 percent (95% CI, 11 to 80%) of neuropathic CPIP patients.

### 3.4. Targeted Muscle Reinnervation

**Figure 3:**
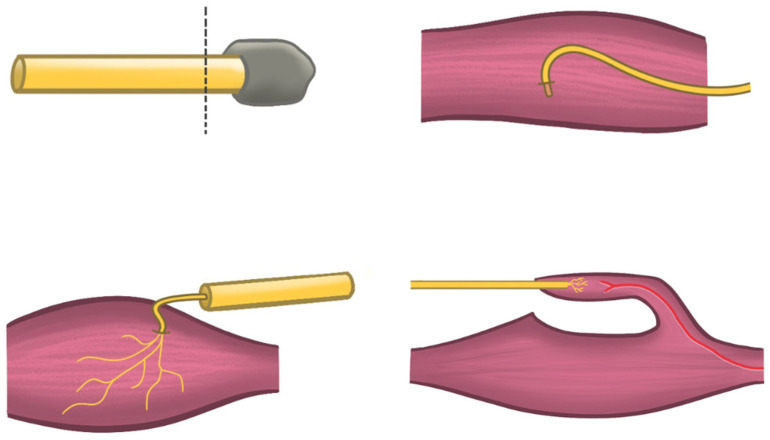
Surgical techniques for neuropathic pain management; neurectomy (**left**), targeted muscle reinnervation, relocation in muscle (**right**), vascularized regenerative peripheral nerve interfaces.

Targeted muscle reinnervation (TMR) was described by Chappell et al., as a surgical treatment for painful abdominal wall neuromas [23]. No other studies reported outcomes on TMR. TMR involves excising the diseased nerve segment and connecting it to a motor nerve serving a functionally expandable muscle nearby (Figure 3). Among the eight patients included in the study, TMR of the ilioinguinal (eight), iliohypogastric (one), and genitofemoral (one) nerves achieved a good postoperative result in 88 percent of patients. The reinnervation was directed towards a motor branch of the internal oblique muscle.

### 3.5. Complications

Regarding complications, seven studies reported no intraoperative or postoperative complications, while three studies documented minimal occurrences of postoperative complications such as surgical-site infection and testicular complications [16,17,21].

## 4. Discussion

Inguinal hernia repair surgery is one of the most performed surgeries worldwide; nonetheless, CPIP and neuropathic CPIP continue to be frequently reported and represent serious adverse outcomes. The precise prevalence rates of neuropathic CPIP remain unclear in the existing literature. However, our literature review found an estimated prevalence of neuropathic CPIP of 53 percent (95% CI, 32 to 73%) among patients with CPIP (Table 2) [7,24,25,26,27,28,29,30,31]. The majority of the studies found no significant association between neuropathic CPIP and the initial surgical approach for hernia repair (open versus laparoscopic) [7,25,26,27,28,29,31].

This systematic review aimed to evaluate the effectiveness of the surgical techniques utilized in managing neuropathic CPIP and distinguish whether one technique is the superior option. Three surgical techniques were identified: selective neurectomy, triple neurectomy, and targeted muscle reinnervation. The proportions of good postoperative results ranged between 46 and 88 percent across these techniques. Overall, surgical treatment of neuropathic CPIP achieved a good postoperative result in 68 percent (95% CI, 49 to 82%) of cases. Targeted muscle reinnervation yielded the highest proportion of good postoperative results; however, this outcome was confined to a singular study with a small group of patients.

The accurate diagnostic assessment of neuropathic CPIP is crucial for patient selection, yet no established protocol exists for this purpose. In this systematic review, the diagnosis of neuropathic CPIP has predominantly relied upon a comprehensive approach, combining clinical signs, physical examination, nerve blocks, and imaging modalities. Typical clinical signs of neuropathic CPIP involve transient electrical stabbing or burning sensations, occurring spontaneously or post-provocation [15]. In contrast, non-neuropathic pain or nociceptive pain frequently manifests as persistent tenderness or pounding sensations, resulting from tissue reactions to inflammatory processes induced by the operation, mesh-related fibrosis, or postoperative fibrosis [7,15,18,29]. Clinical signs for neuropathic CPIP are mainly assessed using screening tools of high specificity for neuropathic pain, such as the Neuropathic Pain Questionnaire (NPQ) and the Douleur Neuropatique 4 Questionnaire (DN4). However, the primary diagnostic method remains a physical sensory examination, aimed at confirming abnormal sensory responses and identifying patterns indicative of nerve injury. Additionally, diagnostic nerve blocks can be used to exclude central pain syndromes and may help to identify the affected nerve(s) and gauge potential response to the surgery [16,20,32]. Imaging modalities are utilized to exclude alternative diagnoses.

The optimal surgical treatment for neuropathic CPIP remains a topic of debate in the current literature. Selective neurectomy refers to the surgical removal of the nerve(s) that is directly associated with the patient’s neuropathic pain while preserving the unaffected nerves to avoid unnecessary risks of deafferentation. During the procedure, any prosthetic material, neuroma, or fibrotic encasement is excised, and the nerve end is cauterized and buried in the internal oblique muscle or allowed to retract to the retroperitoneum [14,18]. In this systematic review, selective neurectomy yielded overall good results in 72 percent (95% CI, 46 to 89%) of neuropathic CPIP patients [14,15,16,17,18]. Triple neurectomy, on the other hand, aims to address all three inguinal nerves at once with the assumption that these nerves are collectively responsible for chronic pain through anatomic variability and cross-innervation [21,22]. Triple neurectomy yielded overall good results in 73 percent (95% CI, 48 to 88%) of neuropathic CPIP patients [21,22]. Selective and triple neurectomy, despite being the most commonly used surgical techniques, lack a physiological target for nerve regrowth. The relocation of the nerve stump into nearby tissues is solely aimed at protecting it and preventing irritation. However, it does not provide a specific physiologic target for regrowth of the nerve since the recipient muscle is already innervated (Figure 3). Consequently, the regeneration of the nerve stump often leads to the formation of a recurrent neuroma, failure to improve pain, and the need for secondary surgery [32]. The study by Zacest et al. reported a recurrence of pain in 68% of patients after the selective neurectomy of the ilioinguinal nerve [17]. In targeted muscle reinnervation (TMR), the neuroma is excised, and the residual nerve stump is coaptated to a motor nerve branch supplying a portion of functionally expendable muscle in the vicinity of the nerve, to allow for reinnervation (Figure 3) [32,33]. The underlying rationale is that providing a clear, physiological normal function for the transected nerve ending helps prevent disorganized growth, hypersensitivity, and recurrent neuropathic pain [32,33]. TMR demonstrated good postoperative results in 88 percent of patients, yielding the highest proportion of good postoperative results [23]. Nevertheless, it is important to note that this conclusion is derived from a single study.

In the field of peripheral nerve surgery, neurectomy has lost its position as the standard surgical technique for symptomatic neuromas. Neurectomy has resulted in only modest pain relief and resection alone seems to be associated with unacceptable recurrence rates of painful neuromas [34]. This can be attributed to the technique’s “passive” nature, as it fails to address the regenerative potential of the nerve stump or provide a pathway for nerve regrowth [32]. According to Chappel et al., optimal management should entail “active” treatment of the excised nerve end, instead of burying or hiding it [23]. Active treatments include vascularized regenerative peripheral nerve interfaces (RPNI) and the previously mentioned targeted muscle reinnervation (TMR). In the case of vascularized RPNI, the excised nerve is implanted into vascularized, free muscle grafts, serving as denervated targets for axon ingrowth from the injured nerve (Figure 3). This approach has exhibited promising clinical results in improving neuroma pain and phantom pain. TMR, which has undergone more extensive research, has reported good postoperative results ranging from 67 to 93 percent [35,36,37]. A recent meta-analysis revealed that TMR achieved good postoperative results in 82 percent of patients with peripheral nerve neuromas, versus 60 percent after neurectomy (*p* < 0.05) [33].

The encouraging results of TMR regarding neuropathic CPIP in this systematic review and the promising outcomes in peripheral nerve surgery suggest a potential paradigm shift towards a more active surgical approach in the treatment of neuropathic CPIP. Future studies, including comparative studies and well-designed randomized controlled trials, are needed to further evaluate and compare the surgical techniques in the management of neuropathic CPIP. Additionally, these studies should explore the potential advantages of TMR and vascularized RPNI regarding neuropathic CPIP.

While this systematic review provides valuable insights into the surgical management of neuropathic CPIP, there are limitations. One significant challenge lies in the heterogeneity observed in the results and reported percentages of neuropathic CPIP across the selected studies. This systematic review is mainly hindered by the small number of available studies, primarily observational, with a lack of randomized clinical trials and a limited inclusion of control groups. Additionally, two included studies did not differentiate between selective and triple neurectomy when reporting outcomes, precluding separate analyses [18,19]. Another limitation relates to the inclusion of studies involving surgeries in the inguinal area other than inguinal hernia repair. Although this decision was justified based on the larger patient population in the inguinal hernia group and the similarity in involved inguinal nerves, it still introduces heterogeneity within the surgical procedures examined. Furthermore, the inclusion of diverse assessment tools, such as ordinal and numerical pain scales, coupled with variations in preoperative and postoperative measures, introduces challenges when comparing the data. To address this limitation, this review adopted a standardized primary outcome—a good postoperative result—to enable comparability across the studies. However, the subjective nature of pain assessment remains a potential source of bias in this review.

## 5. Conclusions

This systematic review investigates the effectiveness of selective neurectomy, triple neurectomy, and targeted muscle reinnervation as surgical interventions in managing neuropathic chronic postherniorrhaphy inguinal pain (CPIP). The surgical treatment of neuropathic CPIP is generally considered safe and effective in postoperative pain relief, yielding good postoperative results in 68 percent (95% CI, 49 to 82%) of neuropathic CPIP patients. Given the substantial prevalence of neuropathic CPIP and its impact on quality of life, the implementation of optimal surgical treatment is essential. Notably, targeted muscle reinnervation has exhibited the highest proportion of good postoperative results in this review and holds promise for surpassing current success rates in inguinal hernia repair surgery. Future well-designed studies are needed to validate these findings and explore active surgical approaches that offer the excised nerve a physiologic target for regrowth.

## Figures and Tables

**Table 1:** Surgical treatment outcomes in neuropathic CPIP.

Author, Year, LoE	Surgical Technique	Mesh Removal	Mean Follow-Up Months (Range)	Number of Patients with Neuropathic CPIP	Number of Patients Pain Free Postoperatively (%)	Number of Patients with Good Result Postoperatively (%)
Ducic et al., 2008 [14], VI	Selective neurectomy	NS	12 (3–24)	18	13/18 (72%)	15/18 (83%)
Vuilleumier et al., 2009 [15], VI		Yes	12 (0–34)	43	41/43 (95%)	41/43 (95%)
Loos et al., 2010 [16], VI		Yes	18 (0–18)	49	10/49 (20%)	26/49 (53%)
Zacest et al., 2010 [17], VI		NS	35 (3–108)	18	5/18 (28%)	7/18 (39%)
Karampinis et al., 2017 [18], VI		NS	14 (5–26)	8	3/8 (38%)	5/8 (63%)
Moreno-Egea, 2016 [19], VI	Selective and triple neurectomy	NS	24 (12–48)	16	11/16 (69%)	11/16 (69%)
Gangopadhyay et al., 2020 [20], VI		NS	6 (0–6)	12	2/12 (17%)	2/12 (17%)
Moore et al., 2016 [21], VI	Triple neurectomy	NS	22 (3–36)	62	13/62 (21%)	50/62 (81%)
Bjurström et al., 2017 [22], VI	Triple neurectomy	NS	6 (0–6)	10	2/10 (20%)	5/10 (50%)
Chappell et al., 2021 [23], VI	TMR	NS	19 (2–54)	8	3/8 (38%)	7/8 (88%)

LoE, Level of Evidence; CPIP, Chronic Postherniorrhaphy Inguinal Pain; TMR, Targeted Muscle Reinnervation; NS, Not specified.

**Table 2:** The prevalence of neuropathic CPIP.

Author (Year), LoE	Number of Patients after Hernia Repair	Number of Patients with CPIP	Prevalence of CPIP (%)	Hernia Repair Technique	Mean Follow-Up Months (Range)	Number of Patients with Neuropathic CPIP	Prevalence of Neuropathic CPIP in CPIP Group (%)
Cunningham et al., 1996 [24], III	276	29	29/276 (11%)	Bassini, McVay, Shouldice	24 (6–24)	2	2/10 (20%)
Poobalan et al., 2001, [25], VI	226	67	67/226 (30%)	Bassini, Lichtenstein	60 (3–60)	31	31/67 (46%)
Ergonenc et al., 2017, [26], VI	264	61	61/264 (23%)	Lichtenstein	3 (3–24)	45	45/61 (74%)
Bande et al., 2020, [27], VI	1761	239	239/1761 (14%)	Open	4 (4–24)	92	92/239 (39%)
Loos et al., 2007, [28], VI	1766	211	211/1766 (12%)	Lichtenstein, Shouldice, TEP, TAPP	46 (3–300)	72	72/155 (47%)
Kalliomaki et al., 2009, [29], VI	98	76	76/98 (76%)	Lichtenstein, Shouldice, laparoscopic	48 (48–62)	47	47/70 (67%)
Voorbrood et al., 2015, [7], VI	NS	105	NS	Lichtenstein, Shouldice, TEP, TAPP	3 (1.5–7)	37	37/105 (35%)
Beldi et al., 2018, [30], VI	96	31	31/96 (32%)	Open, laparoscopic	56 (12–76)	9	9/31 (29%)
Oliveira et al., 2018, [31], VI	829	199	199/829 (24%)	Open, laparoscopic	NS	75	75/199 (38%)

LoE, Level of Evidence; CPIP, Chronic Postherniorrhaphy Inguinal Pain; NS, Not Specified.

## Appendix A. Search Results and Search Terms

**Database Searched**	**Platform**	**Years of Coverage**	**Records**	**Records after Duplicates Removed**
Medline ALL	Ovid	1946–Present	2328	2309
Embase	Embase.com	1971–Present	3135	1079
Web of Science Core Collection	Web of Knowledge	1975–Present	1973	459
Cochrane Central Register of Controlled Trials	Wiley	1992–Present	1306	184
Additional Search Engines: Google Scholar			200	36
Total			8942	4067

### Appendix A.1. Medline ALL Ovid 2328

(Hernia, Inguinal/OR (Groin/AND (Herniorrhaphy/)) OR (((inguin* OR ilioinguin* OR groin*) ADJ3 herni*) OR Lichtenstein*).ab,ti.) AND (Neuralgia/OR Nerve Compression Syndromes/OR Pain, Postoperative/OR (neuropath* OR (nerv* ADJ3 (injur* OR compress*)) OR ((postherni* OR post-herni* OR postoperat* OR post-operat* OR postsurg* OR post-surg*) ADJ3 pain*) OR (pain* ADJ6 (repair*)) OR (target* ADJ3 (muscle OR muscular*) ADJ3 (reinnervat* OR re-innervat*))).ab,ti. OR (pain* AND (repair*)).ti.) AND english.la. NOT (exp animals/NOT humans/)

### Appendix A.2. Embase.com 3135

(‘inguinal hernia’/de OR (‘inguinal pain’/de AND (herniorrhaphy/de OR hernioplasty/de)) OR ‘Lichtenstein method’/de OR ‘lichtenstein technique’/de OR ‘lichtenstein repair’/de OR ‘lichtenstein hernioplasty’/de OR ‘lichtenstein procedure’/de OR (((inguin* OR ilioinguin* OR groin*) NEAR/3 herni*) OR Lichtenstein*):Ab,ti) AND (neuropathy/de OR ‘neuropathic pain’/de OR ‘nervous system injury’/de OR ‘nerve injury’/de OR ‘nerve compression’/de OR ‘postoperative pain’/de OR ‘targeted muscle reinnervation’/de OR (neuropath* OR (nerv* NEAR/3 (injur* OR compress*)) OR ((postherni* OR post-herni* OR postoperat* OR post-operat* OR postsurg* OR post-surg*) NEAR/3 pain*) OR (pain* NEAR/6 (repair*)) OR (target* NEAR/3 (muscle OR muscular*) NEAR/3 (reinnervat* OR re-innervat*))):ab,ti OR (pain* AND (repair*)):ti) AND [english]/lim NOT ([conference abstract]/lim AND [2000–2019]/py) NOT ([animals]/lim NOT [humans]/lim)

### Appendix A.3. Web of Science 1973

TS=(((((inguin* OR ilioinguin* OR groin*) NEAR/2 herni*) OR Lichtenstein*)) AND ((neuropath* OR (nerv* NEAR/2 (injur* OR compress*)) OR ((postherni* OR post-herni* OR postoperat* OR post-operat* OR postsurg* OR post-surg*) NEAR/2 pain*) OR (pain* NEAR/5 (repair*)) OR (target* NEAR/2 (muscle OR muscular*) NEAR/2 (reinnervat* OR re-innervat*))))) NOT DT=(Meeting Abstract OR Meeting Summary) AND LA=(english)

### Appendix A.4. Cochrane CENTRAL 1306

((((inguin* OR ilioinguin* OR groin*) NEAR/3 herni*) OR Lichtenstein*):Ab,ti) AND ((neuropath* OR (nerv* NEAR/3 (injur* OR compress*)) OR (pain* NEAR/3 (repair* OR postoperat* OR post-operat* OR postsurg* OR post-surg*)) OR (target* NEAR/3 (muscle OR muscular*) NEAR/3 (reinnervat* OR re-innervat*))):ab,ti)

### Appendix A.5. Google Scholar

“inguinal|ilioinguinal|groin hernia|herniorraphy” neuropathy|neuropathies|”nerve injury|injuries”|compression|”postherniorraphic|postoperative|postsurgical pain”|”targeted muscle|muscular reinnervation”

## Appendix B. Classification of Strength of Evidence by Jovell and Navarro-Rubio [15]

**Level**	**Strength of Evidence**	**Type of Study Design**
I	Good	Meta-analysis of randomized controlled trials
II		Large-sample randomized controlled trials (*n* > 25 for each group)
III	Good to fair	Small-sample randomized controlled trials (*n* < 25 for each group)
IV		Non-randomized controlled prospective trials
V		Non-randomized controlled retrospective trials
VI	Fair	Cohort studies
VII		Case-control studies
VIII	Poor	Noncontrolled clinical series; descriptive studies
IX		Anecdotes or case reports

## Appendix C. Quality Assessment Using the NIH Tool for Observational Cohort and Cross-Sectional Studies [13]

**Study**	**Q1**	**Q2**	**Q3**	**Q4**	**Q5**	**Q6**	**Q7**	**Q8**	**Q9**	**Q10**	**Q11**	**Q12**	**Q13**	**Q14**	**Rating**
Ducic et al. [14] (2008)	+	+	−	+	−	?	+	?	+	−	?	?	+	?	Fair
Vuilleumier et al. [15] (2009)	+	+	−	+	+	+	+	?	+	+	+	?	+	?	Good
Loos et al. [16] (2010)	+	+	−	+	+	+	+	?	+	+	+	?	+	?	Good
Zacest et al. [17] (2010)	+	+	−	+	+	+	+	?	+	+	+	?	+	?	Good
Karampinis et al. [18] (2017)	+	+	−	+	+	+	+	+	+	+	+	+	+	+	Good
Moreno-Egea et al. [19] (2016)	+	+	−	+	+	+	+	+	+	+	+	?	+	+	Good
Gangopadhyay et al. [20] (2020)	+	+	−	+	+	+	+	+	+	+	+	?	+	?	Good
Moore et al. [21] (2016)	+	+	−	+	+	+	+	+	+	+	+	?	+	?	Good
Bjurström et al. [22] (2017)	+	+	−	+	+	+	+	+	+	+	+	?	+	?	Good
Chappel et al. [23] (2021)	+	+	−	+	+	+	+	?	+	?	+	?	+	?	Fair
+: Yes; −: No; ?: Unclear.															

- Q1: Was the research question or objective in this study clearly stated?
- Q2: Was the study population clearly specified and defined?
- Q3: Was the participation rate of eligible persons at least 50%?
- Q4: Were all the subjects selected or recruited from the same or similar populations (including the same time period)? Were inclusion and exclusion criteria for being in the study prespecified and applied uniformly to all participants?
- Q5: Was a sample size justification, power description, or variance and effect estimates provided?
- Q6: For the analyses in this study, were the exposures of interest measured prior to the outcome(s) being measured?
- Q7: Was the time frame sufficient so that one could reasonably expect to see an association between exposure and outcome if it existed?
- Q8: For exposures that can vary in amount or level, did the study examine different levels of the exposure as related to the outcome (e.g., categories of exposure, or exposure measured as a continuous variable)?
- Q9: Were the exposure measures (independent variables) clearly defined, valid, reliable, and implemented consistently across all study participants?
- Q10: Were the exposures assessed more than once over time?
- Q11: Were the outcome measures (dependent variables) clearly defined, valid, reliable, and implemented consistently across all study participants?
- Q12: Were the outcome assessors blinded to the exposure status of participants?
- Q13: Was loss to follow-up after baseline 20% or less?
- Q14: Were key potential confounding variables measured and adjusted statistically for their impact on the relationship between exposures and outcomes?
